# Alternative splicing in prostate cancer progression and therapeutic resistance

**DOI:** 10.1038/s41388-024-03036-x

**Published:** 2024-04-24

**Authors:** Chitra Rawat, Hannelore V. Heemers

**Affiliations:** Department of Cancer Biology, Lerner Research Institute, Cleveland Clinic, Cleveland, OH 44195 USA

**Keywords:** Prostate cancer, Mechanisms of disease

## Abstract

Prostate cancer (CaP) remains the second leading cause of cancer deaths in western men. CaP mortality results from diverse molecular mechanisms that mediate resistance to the standard of care treatments for metastatic disease. Recently, alternative splicing has been recognized as a hallmark of CaP aggressiveness. Alternative splicing events cause treatment resistance and aggressive CaP behavior and are determinants of the emergence of the two major types of late-stage treatment-resistant CaP, namely castration-resistant CaP (CRPC) and neuroendocrine CaP (NEPC). Here, we review recent multi-omics data that are uncovering the complicated landscape of alternative splicing events during CaP progression and the impact that different gene transcript isoforms can have on CaP cell biology and behavior. We discuss renewed insights in the molecular machinery by which alternative splicing occurs and contributes to the failure of systemic CaP therapies. The potential for alternative splicing events to serve as diagnostic markers and/or therapeutic targets is explored. We conclude by considering current challenges and promises associated with splicing-modulating therapies, and their potential for clinical translation into CaP patient care.

## Introduction

Prostate cancer (CaP) remains a significant health problem. In the United States alone, CaP is expected to cause the deaths of more than 35,250 men in 2024 [1]. This mortality is due to acquired resistance to the systemic therapies for metastatic CaP. For more than eight decades, such treatments have hinged on interference with the action of the androgen receptor (AR), a ligand-activated transcription factor that is a major driver of CaP progression [2]. For the majority of castration-resistant CaPs (CRPC) that re-emerge under AR-targeting androgen deprivation therapy (ADT), growth continues to depend on AR. In a subset of up to 20% of CRPC cases, most often referred to as neuroendocrine CaP (NEPC), potent ADT drugs lead to AR-indifference and the emergence of neuroendocrine, stem cell-like or other phenotypes [3–7]. Resistance to ADT results from diverse genomics, transcriptomics and epigenomics mechanisms, several of which lead to restoration of AR activity. Analyses on CRPC specimens have confirmed that ADT induces alterations that impact the gene encoding AR (e.g. gene amplification, gain of function somatic mutations) [8–10] or the biochemical pathways that control androgen biosynthesis and metabolism (e.g. deregulated expression of steroidogenic genes such as *CYP17A1, AKR1C3, HSD17B3, HSD3B2, SRD5A1, SRD5A2*) [11–13]. Other alterations such as PTEN loss in localized ADT-naïve CaPs also influence a patient’s response to ADT and contribute to CRPC progression [14]. Development of NEPC is similarly marked by specific genomic and transcriptomics events (e.g. loss of p53, Rb) [15, 16] and epigenome changes (such as hypomethylation of histone H3 by SOX2 or EZH2 [17, 18]). Several of these alterations are under investigation as biomarkers of ADT response or as novel targets for CaP therapy after failure of ADT.

**Fig. 1:**
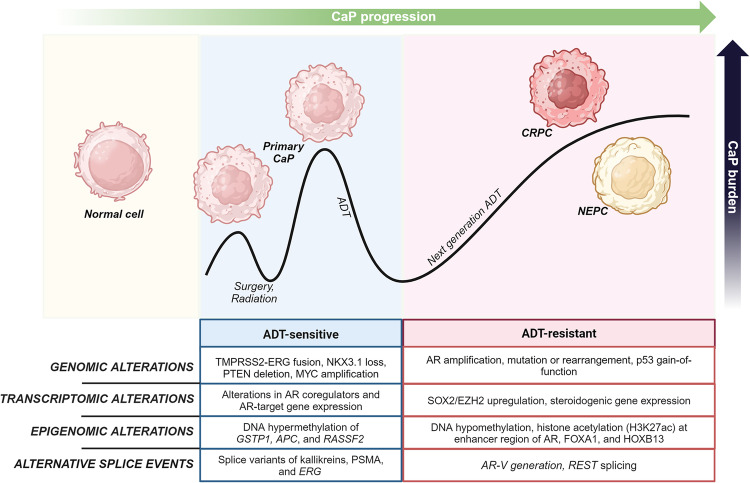
Genomics, transcriptomics and epigenomics alterations during the progression to advanced treatment-resistant CaP. Localized treatment-naïve CaP progresses to CRPC and, in some cases, to NEPC. Each CaP stage and transition is characterized by recurring genomic, transcriptomics and epigenomics changes, for which representative examples are listed. A growing body of evidence supports that shifts occur also in the alternative splicing landscape as CaP progresses and develops resistance to ADT. Such recurring alternative splicing events are listed. ADT, androgen derivation therapy, CaP, prostate cancer; Surgery and radiation, standard of care treatments for localized untreated CaP. The figure was generated using Biorender.

However, the impact of alternative splicing, which has been recognized recently as a hallmark of CaP aggressiveness [19], in the development of treatment resistance and aggressive CaP behavior has not yet been fully explored. Nonetheless, several alternative splicing events have been identified as determinants of the emergence of both major types of late-stage treatment-resistant CaP, i.e. CRPC and NEPC. Notable examples include AR splice variants that emerge under ADT and result in loss of a functional AR ligand-binding domain that is required for binding of ADT drugs and thus cause failure of ADT [20, 21]. Moreover, alternative splicing of REST that is mediated by the spliceosome component SRRM4 promotes neuroendocrine differentiation of CRPC cells and development of NEPC [22–24] (Fig. 1). Recent multi-omics analyses of clinical CaP specimens have started to uncover the landscape of alternative splicing events, revealing numerous gene transcript isoforms that can influence diverse aspects of CaP biology and evolve during CaP progression [19, 25, 26]. Despite their relevance to CaP progression and treatment failure, the molecular basis for the induction of alternative splicing events during CaP growth is poorly understood. How the resulting transcripts differentially affect CaP cell behavior also remains largely unknown, and therapeutic strategies to reverse their effects are not available. Such insights are essential if we are to develop effective approaches to overcome splicing-induced acquired treatment resistance and to reduce CaP mortality. Here, we review literature to better define and understand the contribution of alternative splicing to CaP growth and treatment failure.

## Molecular basis and regulation of alternative splicing

### Major types of alternative splicing events

Splicing entails the removal of non-coding introns and the precise joining of exons during the conversion of a pre-mRNA transcript to a fully mature mRNA [27]. Alternative splicing is a splicing process in which a single gene, via alternative use or specific combination of exons and introns, can produce different distinct mature mRNAs, and consequently, a diverse array of protein products in which specific functional domains are included or omitted or which are subject to frame shifts [27]. The resulting proteins can thus differ markedly in cellular functions. It has been estimated that over 95% of genes are subjected to alternative splicing [28] and that on average seven transcript isoforms are generated from a human gene, suggesting 150,000 transcript isoforms for the 20,000 protein coding genes [29]. Alternative splicing can thus greatly expand the functional diversity encoded by the human genome [27].

Alternative splicing takes different forms depending on how exons are combined. Five major types of alternative splicing events have been identified [30]. In skipped exon (SE) events, different exons are omitted during the transformation of a pre-mRNA to mRNA transcript. Alternative 5′ splice site (A5′SS) and alternative 3′ splice site (A3′SS) events result from alternating splice sites which alter exon lengths while mutually exclusive exons (MXEs) occur when only one of 2 or more adjoining exons is spliced in the mature mRNA. As the name suggests, retained introns (RIs) are the introns which escape removal from pre-mRNA during splicing and are maintained in the synthesis of mature mRNA. While SEs are the most common events in normal mammalian cells [31], in cancer cells, SEs (29%) and RIs (27%) occur most frequently [32].

### Molecular determinants of alternative splicing

**Fig. 2:**
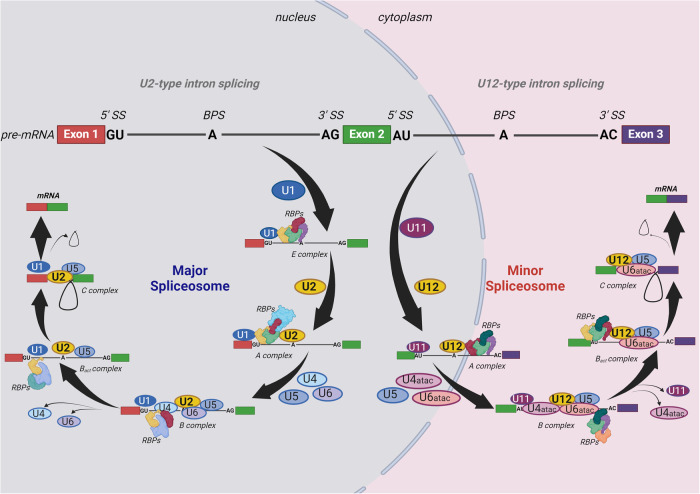
Schematic overview of the mechanisms of action of the major spliceosome and the minor spliceosome. During major spliceosome formation and action, U1 snRNP first recognizes the 5′end splice site (5′SS) of the target pre-mRNA and binds to it along with other non-snRNP splice factors to form the early spliceosome E complex. The non-snRNP SF1 (not shown) recognizes the branch point sequence (BPS) pre-bulging the BPS adenosine for base-pairing with the U2 snRNA, followed by U2AF2 interaction with SF1 (not shown) and recruitment of the U2 snRNP to the spliceosome to form an intermediate A complex. The U4/U5/U6 tri-snRNP is recruited to the spliceosome to form another intermediate complex B. The B complex undergoes extensive conformational rearrangements due to U1 and U4 dissociation and by the action of the RNA helicase DHX16 (not shown) resulting in the formation of the catalytically active Bact complex. The Bact complex catalyzes the first step of splicing, generating the cleaved 5′ exon and intron-3′ exon lariat intermediates and forming complex C. After additional RNP rearrangements, the C complex catalyzes the second step of splicing, resulting in the ligation of the 5′ and 3′ exons and release of the intron in the form of a lariat. The minor spliceosome assembly is similar to that of major spliceosome except that it requires U11, U12 and U4atac/U6atac snRNPs as the functional analogs of the U1, U2 and U4/U6 snRNPs along with the U5 snRNP in the major spliceosome. The figure was generated using Biorender.

The generation of alternative spliced transcripts is tightly regulated by both cis-acting and trans-acting elements. With regard to the latter, splicing occurs via a multimegaDalton dynamic ribonuclear protein complex that is known as the spliceosome. The spliceosome consists of small nuclear RNAs (snRNAs) such as U-rich RNA molecules and dozens of RNA-binding proteins (RBPs), which together with snRNAs make up small nuclear ribonucleoprotein complexes (snRNPs) [33]. An array of non-snRNP splice factors such as RNA-binding motif proteins (RBMs), serine/arginine-rich proteins (SRs), heterogeneous nuclear ribonucleoproteins (hnRNPs) and other proteins are also recruited into the spliceosome [33]. To date, more than 200 proteins have been reported as associated with the spliceosome [34, 35] and to execute the different steps of splicing that have been described before and are summarized in Fig. 2. It is important to note that two types of spliceosomes have been described, a major and a minor spliceosome [36]. The major spliceosome, which is usually what the term spliceosome is taken to refer to, is the first and best characterized type. It is composed of five snRNPs, namely U1, U2, U4, U5 and U6, and catalyzes the splicing of the canonical U2-type introns [37]. The minor spliceosome consists of U5, U11, U12, U4atac, and U6atac snRNPs and controls the splicing of U12-type introns [38, 39]. In vertebrate genomes, the major spliceosome accounts for splicing of 99.5% of introns while the minor spliceosome splices only 0.5% of introns [28]. Some similarities between the two types of spliceosomes have emerged also. For instance, it is now clear that the minor spliceosome can also catalyze some U2-type introns [40]. While it is well-known that major splicing occurs in the cell nucleus, there are some contradictory reports on the localization of minor spliceosomal snRNAs. Some [41] reported the presence of U11 and U12 snRNAs in nucleus of mouse tissues and human cells, while others [42] showed that U12 and U6atac were present in the cytoplasm of multiple zebrafish tissues. Another study [43] also demonstrated that the splicing of the minor-intron gene P120 occurs exclusively in the nuclear compartment of the Xenopus oocyte. A gene can also contain both U2 and U12-type introns and undergo major and minor splicing, respectively, which can regulate alternative splicing of the same gene independently [44, 45].

### Finetuning of alternative splicing output

Splicing is a well-controlled, multi-step process that is achieved by the stepwise assembly of snRNAs and RBPs on cis-acting elements in the pre-mRNA transcript (Fig. 2). The snRNPs are recruited to the 5′ splice site (5’SS) and to the branch point sequence (BPS) with contains an adenosine moiety for base-pairing with the U2 snRNA, while the non-snRNP proteins, RRMs, SRs, hnRNPs and other trans-acting factors bind to the polypyrimidine tract (PPT), located about 5–40 base pairs before the 3′ splice site (3′SS).

Other cis-regulatory elements such as 5′SS, 3′SS, exonic splicing enhancers (ESEs) or silencers (ESSs) and intronic splicing enhancers (ISEs) or silencers (ISSs) in the pre-mRNA sequence also play important roles in the recognition of exon-intron junctions that result in the formation of accurate mRNA transcripts [46]. The strength of these elements, i.e. the extent/affinity of their interaction with trans-acting factors that bind them, along with the abundance of these interacting proteins, determine the exon/introns that are retained or excised in the mature transcript. These elements exhibit evolutionary relationships which may be “compensatory” where one element compensates the weakening of other element by strengthening itself or “correlated” where strengthening of one element is associated with strengthening of the other element. For example, G-rich sequences in the ISEs with at least one G-triplet promoted exon skipping by attracting hnRNP H and F to downstream introns and are reliant on the strength of the adjacent 5′SS for their action [47]. Via computational modeling, it was noted also that while the strength of the 5′SS and the strength of numerous classes of ESSs have evolved in a correlated manner, some ESEs are evolved in a compensatory fashion relative to the 5′SS and 3′SS [48]. However, several aspects of the role of cis-acting elements are still incompletely understood. The molecular basis whereby the same combination of the same regulatory sequence motif and RBPs can lead to context-dependent effects remains unclear [49, 50]. Similarly, the precise composition and contribution of sequences nearby the splicing site that have been recognized to impact the resulting variant transcripts need to be resolved [51]. There is also some debate as to what extent alternative splicing patterns are subjected to co-transcriptional regulation, in which the rate of transcriptional elongation impacts the time during which alternative splice sites are available and can be selected or omitted [52, 53]. Nonetheless, it is obvious that a complex interplay between RNA-RNA, protein-RNA and protein-protein interactions defines exon/intron boundaries, dictates the different forms of alternative splicing and the composition of resulting mRNA variants. Other than the factors mentioned already, additional levels of regulation come into play when defining the protein isoform spectrum. Disrupting the sequence integrity of the many spliceosome proteins and splicing factors that are each involved in different steps of the splicing process can alter the final spectrum of transcripts that are expressed in a cell. Mutations in the splicing-regulatory genes (SRGs) may cause spliceosomopathies that result in dysregulation of splicing and are associated with multiple cancer types, particularly in myelodysplastic syndrome [54, 55] and other hematological malignancies [56–58] but also in solid tumors such as uveal melanoma [59–61], lung cancer [62], breast cancer [63], and pancreatic cancer [64].

Often underestimated are upstream events such as the signal transduction that influences the post-translational modifications of SRGs. In particular, the phosphorylation or dephosphorylation of spliceosome components and splicing factors by multiple classes of kinases (SRPKs, AKT, DYRKs, and CLKs) and phosphatases (PP1 and PP2) has a major impact on their ability to bind RNA, form effective complexes and execute key alternative splicing steps. The best studied is the SR-protein kinase SRPK1 which is highly expressed in many tumors such as cancers of breast, pancreas, lung, colon, prostate and gliomas [65]. Silencing of SRPK1 in cancer cells induces altered VEGF splicing to antiangiogenic VEFG_165_b isoform, enhances apoptosis, and decreases migration and invasion [66–69]. In addition to SRPK1, other kinases have also been shown to regulate alternative splicing by phosphorylating SRG proteins. For example, Cdc2-like kinase 1 (Clk1) phosphorylates the spliceosome protein SPF45 at eight serine residues (serines 48, 62, 202, 204, 222, 266, 288 and 291) inducing exon 6 exclusion from the death receptor mRNA, generating a secreted dominant-negative Fas protein and stimulating ovarian cancer cell migration and invasion [70].

## Alternative splicing events in CaP

### Alternative splicing events have biological consequences

Alternative splicing is important during development and tissue- and cell-specific splicing patterns have been reported [71, 72]. Yet to what extent alternatively spliced transcripts differentially impact cell behavior is not always clear and has been a topic of debate. It has indeed been accepted that some of these events represent splicing errors and may be non-functional transcripts [73]. Alternative splicing is known to also alter the manner in which RNA is processed, as well as RNA stability and localization. In fact, nonsense mediated decay (NMD) has been linked to alternate splicing, whereby changes in alternative splicing contribute to RNA degradation via NMD [74]. On the other hand, several alternatively spliced transcripts have been clearly implicated in establishing tissue-specific expression patterns, to have functional consequences and to lead to changes in cell behavior [75–77].

Diverse mRNA isoforms have been found to have causal roles also in the etiology of human disorders and diseases such as neurodegenerative diseases [78–80], and neuropsychiatric disorders [81]. Notably, compared to their matching normal adjacent tissues, many human cancers are characterized by shifts in the spectrum of transcripts, which are associated with either worse patient survival or increased disease severity, or have been linked with CaP racial disparities that are known to impact its aggressiveness [82–84]. In hematologic cancers in particular, the contribution of these splicing alterations to treatment failure and cancer growth has been well established and has led to novel therapeutic modalities such as SF3B1 and RBM39 inhibitors that target the underlying molecular events [85–87].

### The spectrum of alternative splicing events shifts during prostate carcinogenesis

In CaP, discrepancies between the composition of cellular proteomes and transcriptomes have long been recognized [88–92]. In studies that compared the results of immunoblotting and transcriptomics assays on the same tissues, only 54.7% and 66.3% performance concordance was noted among protein and transcript levels between benign and localized CaP and between localized treatment-naïve and metastatic CaP, respectively [92]. Similarly, extensive proteogenomic analyses of intermediate risk treatment-naive localized CaP have revealed that mRNA abundance changes explain only 10% of protein abundance variability [90]. Whether, and the extent to which, these discrepancies relate to diversity in transcript isoforms could not be assessed. Isolation of some of the first alternatively spliced transcripts, for instance information on those for kallikrein (hK2) dates back to late 1990s and yielded information that soon after was used to develop assays detect metastatic CaP [93]. Since then, candidate transcript approaches have identified several other isoforms that impact genes encoding, for instance, PSMA, PSA, and cyclin D1 in CaP [94–97].

### Alternative splicing events associate with CaP progression and outcome

More recently, the spectrum of alternative spliced transcripts in CaP clinical specimens and their evolution during the different steps of CaP progression and treatment resistance has been explored in a more focused manner. In an early study, investigators who used exon-junction microarray-based assays to profile 1532 mRNA splice isoforms from 364 potential CaP-related genes in 38 localized CaP tissues found that isoform information was better at predicting the presence of CaP than a classifier that considered overall mRNA abundance [98]. This work supported that additional clinically relevant information can be derived from examining alternative splicing events.

To date, several groups have obtained transcript information from RNA-Seq datasets that they either generated themselves or that were available in the public domain. It is important to note that the majority of these studies have been performed on treatment-naive tissues often with normal prostate tissues as controls or reference. The number of CaP specimens that were studied ranged from 14 to over 500 [99, 100] and each study focused on identifying differentially expressed CaP-specific transcript isoforms. Other recurring findings among these studies include that transcript isoforms whose expression was significantly altered were not accompanied by differential expression at the overall mRNA level for the same gene, indicating specific roles for transcripts in CaP progression [98]. When the Cancer Genome Atlas (TCGA) Splice Seq database was examined for transcripts that are relevant to prognosis, overall survival, disease-free survival or progression-free survival by different teams, little overlap was found between their results [98–105]. The reason for these differences was that each study asked a different question and correlated these events with distinct clinical features such as overall survival, disease-free survival, recurrence-free survival, progression-free survival or metastasis. Their results do indicate that CaPs can be classified based on alterative transcripts. Pathway analyses such as KEGG on the differentially expressed transcripts returned processes such as fatty acid metabolism and oxidative phosphorylation [104, 105], which have been independently linked to CaP progression. Other analyses suggested differential splicing patterns can predict CaP outcomes, therapeutic responses or metastatic progression [99, 101, 102, 104]. For instance, such patterns correlated with immune cell infiltration indicating the possibility of differential response to immune therapeutics based on alternative splicing landscape. Differential splicing patterns in CaPs from African-American (AA) compared to Caucasian-American (CA) patients may help explain the more aggressive CaP biology and drug resistance in AAs [26]. Another study compared RNA-Seq datasets directly, verified the evolution in expression of RNA isoforms during CaP progression and under treatment and compared this in localized CaP and benign tissues, in treatment-naïve CaP and in CaP undergoing neoadjuvant ADT, in treatment-naïve CaP and in CRPC, and in CRPC and NEPC [19]. Overall, the number of differential transcripts increased with each step of CaP progression and after acquired treatment resistance. Notably, the authors also found different sets of RNA variants enriched in NEPC and CPRC, suggesting stage- or phenotype- specific events, which was confirmed using cell lines engineered by RB and p53 loss to transition from CRPC to NEPC [19].

### Alternative splicing events that drive treatment resistance and the emergence of CaP phenotypes

Taken together, data from the above studies support alternative splicing events that mediate clinically relevant processes and are augmented during CaP progression. An important caveat to consider is that not all of these studies validated the results using independent datasets nor explored the impact of transcripts that were returned as differentially expressed on CaP cell behavior. However, a number of independent studies do support a causal role for such transcripts in CaP growth and treatment resistance. Indeed, metabolomics studies indicated that only the HSD17D4 isoform 2 that has been implicated in androgen inactivation is lost in CRPC patients, which facilitates ADT resistance [106]. Similarly, ectopic expression of a FGFR3 splice variant that is preferentially expressed in AA CaP and omits exon 14 that encodes the activation loop in the intracellular split kinase domain in European American (EA) CaP cell lines (PC-3 and LNCaP) causes receptor autoactivation, oncogenic signaling cascades and resistance to docetaxel, a chemotherapeutic used in advanced CaP [107].

The best-known examples of alternative splicing events that dictate treatment resistance and emergence of new CaP phenotypes are those that impact the genes encoding AR and REST. Full-length AR is a nuclear hormone receptor that contains four functional domains; an N-terminal domain (NTD) that harbors a constitutively active transactivation domain, a DNA-binding domain by which its binds its target genes (DBD), a hinge region, and the ligand-binding domain (LBD) that spans its ligand-activated transactivation function. Androgen binding to the LBD leads to AR entry into the nucleus where the DBD binds to the androgen-response elements (AREs) of AR target genes regulating their transcription [108]. Many structurally diverse variants of AR emerge in CaP under the pressure of ADT. These shorter versions generally lack a functional LBD, are constitutively active transcription factors that do not respond to ADT and can result from gene rearrangements or alternative splicing. They are commonly known as AR splice variants. To date, more than 20 AR splice variants have been identified [109], of which a few have been studied in more detail. The most extensively studied is variant AR-V7, which forms a truncated receptor in which exons 4–8 of the full-length AR are omitted, is associated with resistance to ADT in CaP patients [110]. Another well-studied variant, AR-V567es, is characterized by the skipping of exons 5, 6, and 7 in the AR gene and has been also been implicated in CRPC [111]. While earlier studies reported the generation of AR-V567es as a result of gene rearrangement, it was later shown that it can also be generated via alternative splicing [112]. AR-V1 and V9 splice variants also can drive androgen-independent growth in CaP, however, are conditionally active [113]. In addition to these, AR variants containing exon 2b (AR-V3, AR-V4, and AR-V5) are constitutively active and mediate CaP therapy resistance [114].

Another alternative splice event with particular relevance to clinical CaP progression is the splicing of the RE1 silencing transcription factor (*REST*). *REST* acts as a repressor of neurogenesis by suppressing genes involved in neuronal differentiation and is itself regulated via alternative splicing to a truncated form REST4. REST4 is generated by the insertion of a neural-specific micro-exon (exon N) between exon 3 and 4 resulting in premature termination [22]. Unlike canonical REST, REST4 cannot bind to the RE1 silencing element in REST target genes and can also prevent the binding of REST to DNA, inhibiting its function and facilitating neuronal differentiation [115]. In CaP, alternative splicing of *REST* to REST4 is mediated by the spliceosome component SRRM4 and promotes neuroendocrine differentiation of CRPC cells and development of NEPC [22–24]. A recent study showed that *REST* splicing can also be regulated by the minor spliceosome machinery [116].

## Molecular basis for shifts in alternative splicing patterns during CaP progression

### Trans-acting factors

#### Differential expression of trans-acting factors

What causes alterations in CaP splicing patterns and steers their evolution during disease progression? As key determinants of alternative splicing, the involvement of trans-acting and cis-acting regulators of the spliceosome has been examined. With regard to the former, several recent reports indicate their differential expression during CaP initiation and progression [19, 25]. For instance, immunostaining of the splicing factor PSF was elevated in a subset of CaP samples compared to benign prostate tissues and higher PSF levels correlated with shorter cancer-specific survival after surgery and shorter PSA-free survival after ADT [117]. Similarly, immunohistochemical analyses revealed high RBM3 protein expression as an independent marker for poor prognosis (shorter time to biochemical recurrence) after radical prostatectomy [118]. An integrated analysis of whole genome (DNA-Seq), transcriptome (RNA-Seq) and methylome (methylome array) data to predict clinical trajectories of CaP identified four molecular subgroups of CaP patients, among which recurrent duplications associated with increased expression of ESRP1 were observed in an aggressive subgroup of early onset CaPs [119]. The role of the SR repetitive matrix SF, SRRM4, which is overexpressed during NEPC emergence, in promoting neuroendocrine differentiation of CRPC cells and development of NEPC has also been well recognized by several groups. These observations link deregulated expression of trans-acting factors to poor CaP outcome but did not necessarily support causality with alternative splicing events. However, alternative splicing analysis using RNA-sequencing data from the Beltran and Vancouver Prostate Centre (VPC) CaP cohorts showed differences in 24 alternative splicing events between NEPC and prostate adenocarcinoma samples, of which 16 were predicted to be regulated by increased levels of SRRM4 [22]. A more comprehensive study measured mRNA expression of 43 spliceosome components and splicing factors in two CaP sample cohorts and found significant CaP dysregulation of 7 and 19 of these, respectively [25]. Increases in the expression of several of these proteins, i.e. SNRNP200, SRSF3 and SRRM1, which was verified by IHC, correlated with clinical features of aggressive disease (Gleason score, t-stage, metastatic progression), as well as alternative splicing events such as AR-V7 that have been linked to ADT failure. Notably, silencing of SNRNP200, SRSF3 and SRRM1 each not only decreased CaP cell proliferation and migration but repressed the levels of oncogenic splicing events such as those impacting AR-V7, PKM2 and XBP1 transcripts [25]. In addition, loss of these factors re-sensitized ADT-resistant CaP cells to the AR antagonist enzalutamide, supporting that the associate splicing events contribute to treatment resistance.

#### Mutational status of trans-acting factors

Another study made use of publicly available transcriptomics and genomics data to explore the expression and/or mutational status of 274 SRGs [19]. Evaluating SRG status in up to 8 mutational databases derived from either treatment-naive CaP or CRPC, 31-68% or 87-94% of primary CaP and CRPC patients were found to have at least one mutation of one SRG. Notably, the vast majority of these alterations consisted of copy number changes due to gene amplifications (mostly in CRPC) or gene deletions (predominantly in localized CaP). Most SRGs were mutated at very low frequencies, with only 7.3 and 10.6% alterations at a rate of >5% of the TCGA localized CaP and the Stand Up To Cancer (SU2C) CRPC cohorts for instance. This observation is consistent with most CaP genomic alterations occurring in long tail of such changes [120]. Of note, actual point mutations in SRGs, which are known to drive liquid cancers’ progression, were rare in CaP as only 4% of the tumors exhibited alterations in five SRGs (*SF3B1*, *U2AF1*, *GEMIN5*, *TCERG1*, and *PRPF8*) with a low mutation rate ranging from 0.5–1.2%. Upon further examination, it was observed that mutations in SF3B1 primarily clustered around the highly conserved HEAT repeats in the C terminus which can interfere with its 3′SS binding and recognition [120]. SRG amplifications increased and deletions decreased during CRPC progression, and copy number alterations impacting SRG genes did correlate with gain and loss of the respective mRNA expression [19]. In addition, also in this study, dysregulated SRG expression correlated with patient prognosis, and two sets of differentially expressed SRGs that correlated favorably or unfavorably, respectively, with CaP progression were identified. In the TCGA cohort, CaPs characterized by high or low expression of the unfavorable signature showed markedly distinct splicing patterns, with the total number of differential splicing events, particularly IR events, higher in the high expression group. The incidence for genomic amplification of SRGs increased during the transition from treatment-naive CaP to CRPC, for instance from 7 to 17% for *ESRP1* and from 8 to 14% for *KHDRBS*. Moreover, silencing of these SRGs in AR-independent PC3 cells (no other cell line models were used), decreased cell viability, migration and sphere formation and altered the global cellular splicing patterns, supporting that these oncogenic SRG properties are associated with alternative splicing events.

**Fig. 3:**
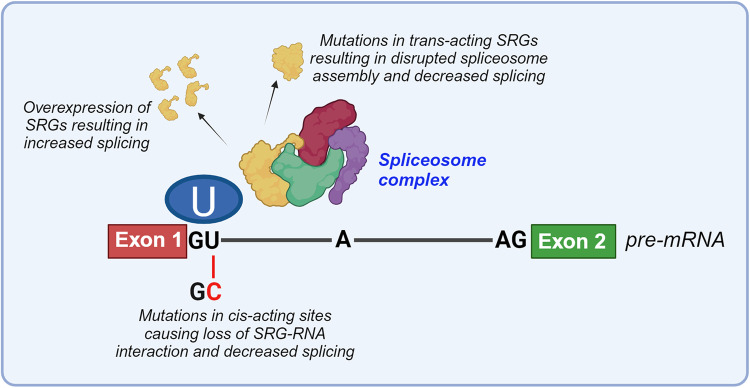
Overview of major molecular determinants that control the spectrum of alternative transcripts that are generated from a pre-mRNA (site). Alternative splicing, and the use of specific splice or regulatory sites, is controlled by both trans-acting factors (top part) and cis-acting factors (bottom part). For example, overexpression of SRGs can stimulate splicing at a receptive sites. Mutations in SRGs on the other hand can result in disruption of the spliceosome and decreased generation of transcripts at receptive sites. Strengths of the cis-acting elements and of their interaction with trans-acting factors can favor the use of specific sites, motifs and thus specific transcript isoforms. Site-specific mutations in RNA sequences can lead to loss of RNA-protein interactions and/or skipping the use of specific site (combinations), resulting in decreased levels of the associated transcripts. The figure was generated using Biorender.

While most of the studies mentioned above focused on components of the major spliceosome, recently a role for minor intron splicing in lethal CaP has been recognized. In this respect, levels of U6atac, a small nuclear RNA belonging to the minor spliceosome, were found to increase during CaP progression in TCGA data and were highest in CRPC cells 22Rv1 and H660 [116]. siRNA-mediated loss of U6atac increased minor intron retention, indicative of defective minor intron splicing, although correlation with the proteome composition was incomplete. Interestingly, minor intron splicing impacted also transcripts of the NEPC lineage-determining factor REST. Silencing U6atac caused cell cycle arrest in cell line and organoid models representing CaP progression and these growth inhibitory effects were more pronounced in CaP compared to benign prostate tissues, suggesting a CaP-specific role for U6atac. Moreover, loss of U6atac outperformed the effect of ADT on growth of primary and CRPC CaP cells. Taken together, these reports do support a causal role for deregulated expression of trans-acting regulators in alternative splicing in CaP (Fig. 3).

#### Mechanisms underlying differential expression and action of trans-acting factors

In view of the common dysregulation of these trans-acting regulators, it is tempting to speculate that mechanisms other than SRG gene amplification and deletions may be at play. Mounting evidence indicates that known drivers of CaP initiation and progression contribute also. An emerging theme is that CaP oncogenes such as AR and other key regulators of CaP growth, including MYC and FOXA1, control expression of one or more SRGs, which then controls distinct alternative splicing patterns [121–127] (Table 1). It should be noted that SRGs may have other ways to contribute therein. Genome-wide CRISPR screens functionally identified spliceosome components and RNA-binding proteins such as HNRNPL as a CaP dependency that regulates RNA splicing, yielding novel molecular insights on SRG relevance to CaP progression [128]. Similarly, by combining an antisense oligonucleotide tiling assay and RNA-binding motif analysis, hnRNP H and hnRNP F were identified as mediating MYC’s control over splicing of a HRAS cassette exon, uncovering new mechanisms for MYC-dependent regulation of splicing [125]. Additional mechanisms may be at play. For one, SRGs themselves are known to control splicing of their own pre-mRNA, suggesting that feedforward mechanisms exist to direct splicing programs [129]. Splicing is well recognized to be tightly controlled by post-translational modifications such as phosphorylation, which determines protein-RNA and protein-protein interactions that are critical for completing each step so the splicing process [130]. In this respect, recent work by our group identified citron kinase, a poorly characterized Ser/Thr kinase that we found to be tumorigenic and to drive CaP growth and treatment resistance, as a key determinant of a novel alternative splicing program that is enriched in CRPC and NECP [131]. Of note, at least one third of CIT’s substrates that we identified consisted of SRGs, suggesting that this kinase coordinately controls a CaP splicing landscape that is relevant to treatment resistance. Another kinase SRPK1, responsible for the phosphorylation of the proto-oncogene splice factor, SRSF1, is also upregulated in CaP, correlating with increased invasion and angiogenesis. In CaP cells, the Wilms tumor suppressor zinc-finger transcription factor (WT1) activates SRPK1 transcription, which can be reversed by the transcriptional corepressor BASP1 leading to increased expression of the antiangiogenic VEGF165b splice isoform [132].

**Table 1:** Drivers of CaP progression control expression or activity of SRGs.

Regulator	Trans-acting factor	Regulation	Conclusion	Reference
AR	ESRP1 and ESRP2	Transcriptional and post-translational	AR controls splicing via regulating mRNA expression and binding of ESRP1/2 around spliced exons	[121, 122]
MYC	hnRNP I, hnRNP A1, and hnRNP A2	Transcriptional	MYC upregulates transcription of hnRNP I, hnRNPA1 and hnRNPA2	[123, 124]
	hnRNP H	Transcriptional	hnRNP H expression is anticorrelated with MYC hallmarks	[125]
	SRSF3	Post-transcriptional	MYC signaling enables SRSF3 to escape from a negative autoregulatory mechanism and stabilize the *SRSF3* mRNA transcript	[126]
FOXA1	hnRNP K and SRSF1	Transcriptional	FOXA1 directly drives the mRNA expression of SRGs, particularly of hnRNP K and SRSF1	[127]

Overview of the impact of AR, FOXA1 and MYC on expression of trans-acting factors under their control.

### Cis-acting factors

Mutations at cis-acting motifs that prevent the interactions with trans-acting factors, which are required to execute the spectrum of alternative transcripts, have been described for other human malignancies. To date, such information is scarce for CaP and in cases where genomic variations are identified, their impact on usability of the site and functional consequences generally not as well established (Fig. 3). Only one study so far has profiled the different splice-disrupt genomic variants in CaP and reported *HLA-A* in primary CaP, *MSR1* in familial CaP, and *EGFR* in both CRPC and metastatic CRPC with the highest allele frequencies of splice-disrupt variations [133]. Nonetheless, several splice-disrupting genomic variants have been predicted during different stages of CaP progression, which impact cancer-relevant genes such as EGFR [133]. The extent to which these predictions hold true is yet to be fully determined. Similarly, GWAS analyses have identified single-nucleotide polymorphisms (SNPs) in stemness-related genes that are predicted to regulate their RNA splicing and demonstrate racial disparity [134]. On the other hand, splicing-specific expression Quantitative Trait Loci (eQTL) analysis revealed a CaP risk SNP that was significantly associated with increased expression of melanophilin (MLPH) variant 4, but not other variants. Variant 4 more strongly promoted cell proliferation and invasion and countered apoptosis than other MLPH variants in CRPC and metastatic CaP cell lines, and was selectively associated with poor recurrence-free survival in TCGA data [135].

## Targeting alternative splicing for CaP treatment

CaP-associated splicing events have been linked to worse outcomes and the emergence of treatment resistance. Therefore, preventing, inhibiting, or reversing their effects has been considered as therapeutic strategy. As key determinants and executors of alternative splicing, both trans- and cis-acting factors have been tested as targets for such approaches - as in other human disorders and malignancies [85, 136–139]. Below we outline some of the tactics that have been explored for CaP treatment, their therapeutic potential and the limitations to moving these forward to clinical practice.

### Trans-acting factors

**Fig. 4:**
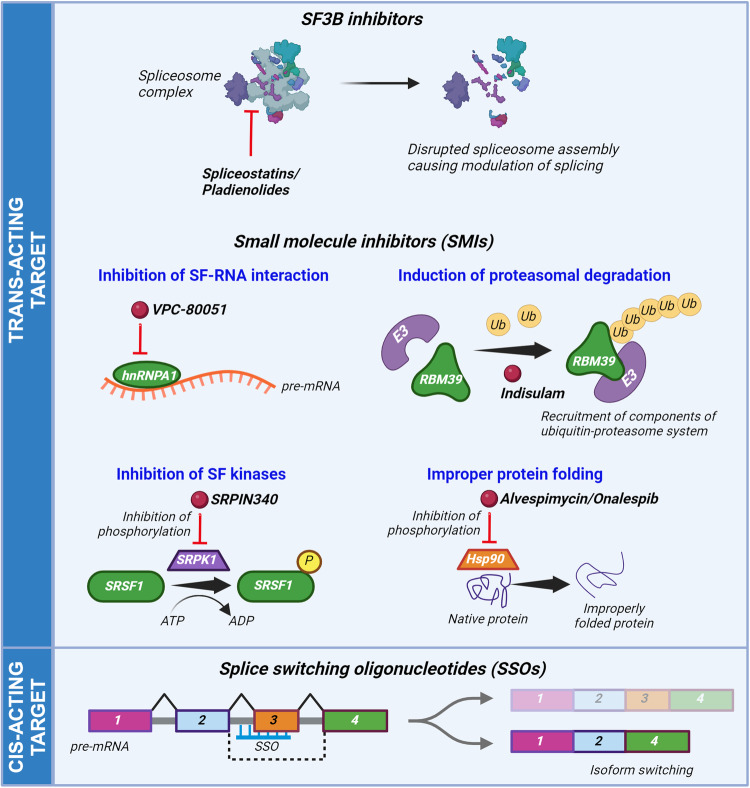
Therapeutic strategies that have been tested in CaP to interfere with alternative splicing. Therapeutic strategies tested in CaP so far have targeted both trans-acting factors (top part) and cis-acting factors (bottom part). The former has involved the preclinical use of inhibitors of spliceosome components such as SF3B and several small molecule drugs/compounds that were designed to inhibit other SRGs. These inhibitors either interfere with splicing factor(SF)-RNA interactions, lead to proteasomal degradation of SRGs, inhibit the activity of kinases that control SF RNA binding and action, or consisted of non-splicing-specific compounds such as Hsp90 inhibitors that were found to impact also CaP-relevant alternative splicing events. Targeting the cis-elements has involved the use of splicing switch oligonucleotides (SSOs) that were designed to target specific pre-mRNA sequences and thereby control the transcript isoforms generated from those sites. The figure was generated using Biorender.

Over the past three decades, several naturally occurring products obtained from bacteria, and their analogs, have been reported to bind to splicing factors and to disrupt spliceosome assembly at the pre-mRNA, thereby causing cytotoxicity in cancer cells. The spliceosome subunit SF3B and its core components has been a major target for such compounds (Fig. 4). To date, three major classes of SF3B inhibitors have been developed, which are known as spliceostatins, pladienolides and herboxidiene [140–142]. The impact of several of these on the growth of preclinical CaP models has been evaluated. For instance, in xenograft models established from SF3B2-overexpressing cells, pladienolide B repressed SF3B2-mediated AR-V7 generation and CaP growth under castration conditions [143]. A derivative of pladienolide B, E7107, which binds specifically to SF3B1 reversed CaP aggressiveness, where the E7107-treated CaP cells showed RNA-Seq-based gene signatures similar to normal prostate tissues but not the cancer tissues, and inhibited CRPC growth in xenograft and autochthonous models [19]. Spliceostatin A inhibited AR-V7 expression potently however causes high in vivo toxicity, which prompted the design of an 1,2-deoxy-pyranose analog [144] and a 4-acetoxypentanamide derivative [145] of spliceostatin A which have less toxicity but same potency. Other compounds that target SRGs were subject to preclinical testing in CaP also. Because AR-V7 confers primary resistance to ADT, SRG-targeting compounds have been designed to target CRPC and tested in CRPC cell line models. For instance, VPC-80051, a first small molecule inhibitor of hnRNPA1 splicing activity was recently discovered using a computer-aided drug discovery approach to target its RNA-binding domain (RBD) [146]. VPC-80051 reduced AR-V7 mRNA levels which correlated with a reduction in viability of in 22Rv1 CRPC cells. However, since these cells also express multiple other constitutively active AR splice variants (e.g. AR-V3 and AR-V4) [147], further investigation is needed to determine the effect of this inhibitor on those variants. In addition, indisulam, a splicing-modulating drug that degrades the splicing factor RBM39 with high selectivity, reduced splicing of AR pre-mRNA and in vivo and in vitro growth of CaP models [148].

Apart from direct inhibitors of SRG activity, compounds that target critical post-translational modifications on several SRGs, and may thus may indirectly impact the activity of more than one SRG, have shown some promise. For instance, small molecule inhibitors of the SRPK1 splice factor kinase that phosphorylates the oncogenic splice factor SRSF1 in CaP cells altered VEGFA splicing to favor the antiangiogenic isoforms [69] and reduce xenograft CaP growth [68]. Other classes of small molecule inhibitors that were originally derived to target different proteins can also act as spliceosome modulators and impact alternative splicing events in CaP. Because the mode of action of these molecules is not splicing-specific, it is not straightforward to determine which activity underlies their growth inhibitory effect. Examples include the HSP90 inhibitors alvespimycin [78] and onalespib [149], which lowered levels of both full-length AR and AR-V7 and delayed CaP xenograft progression. It is assumed that the chaperone protein HSP90 is involved in AR folding and its inhibition targets different AR protein isoforms for proteasomal destruction. Noteworthy, other HSP90 inhibitors increased AR-V7 expression (luminespib, [150]) or had no effect on AR-V7 levels (geldanamycin, [151]). Administration of another type of small molecule inhibitor, the L-type calcium channel antagonist nemadipine-A in a nude mouse xenograft model of CaP slowed tumor growth and in PC3 cells inhibited cell migration and switched FGFR2 splicing [152].

### Cis-acting factors

RNA-based therapeutic strategies that interfere with spliceosome interactions at key determinant cis-acting RNA sequences can correct clinically relevant alternative splicing events in CaP. Such approaches make use of splice-switching oligonucleotides (SSOs), short synthetic antisense nucleic acids that hybridize to pre-mRNA and are intended to bind complementary to regulatory exonic and intronic sequences or exon-intron junctions so relevant SRGs cannot interact there. SSOs targeting the polypyrimidine tract (PPT) region of the original 3′SS of exon 3 (S-3′ SS) of AR strongly enhanced the retention of 23-amino acid long exon 3a and resulted in increased levels of AR23, a splice variant known to block AR entry into the nucleus and restore the sensitivity of CaP cells to ADT in 22Rv1 cells and reduced cell proliferation [153]. Targeting the 5′SS of exon 2 of BCL2L1 pre-mRNA, other SSOs have been used to alter BCL2L1 splicing causing decreased production of the antiapoptotic BCl-XL and increases in the proapoptotic BCL-Xs splice isoform, which led to reduced CaP growth in vivo [154]. Furthermore, SSO-induced Bcl-xS isoform made CaP cells more sensitive to γ-irradiation, UV light, and chemotherapeutic treatments [155]. Another example of a SSO-based therapy was aimed at the splicing location between intron 5 and exon 6 in the telomerase reverse transcriptase hTERT pre-mRNA to modify its splicing pattern in DU145 CaP cells. This strategy caused a decrease in the full-length hTERT transcript and an increase in alternatively spliced transcripts with loss of telomerase catalytic activity, resulting in a significant decrease in cell proliferation and apoptosis activation [156]. The role of ERG splicing in CaP suggested a correlation of a high ratio of full-length ERG type I variant to the truncated type II variant with less favorable outcomes in patients [157]. SSOs that target both the 5′ and 3′SSs of ERG’s exon 4 were shown to induce exon 4 skipping introducing a frameshift stop codon nine bases into exon 5, which resulted in a reduction of overall ERG protein levels, decreased cell proliferation, cell migration and significantly increased apoptosis in in vitro and in vivo model systems [158]. One study also tested SSOs that target the expression of the NEPC-associated trans-acting splice factor SRRM4, which successfully downregulated SRRM4 in 22Rv1 and VCaP cells, modified the alternative splicing of REST, and resulted in reduced cell viability. The authors also tried a SSO-based strategy targeting REST, which yielded similar anti-proliferative effects [159].

#### Limitations to current approaches

Even though cis-acting and trans-acting targeting approaches each have shown therapeutic potential, it is important to point out the limitations, caveats and gaps in knowledge that will need to be addressed.

How the spectrum of alternative transcripts changes under standard of care treatments for metastatic CaP has not yet been fully resolved but is critical information to move forward with target selection for the proposed therapies. A couple of groups have reported that androgens, via AR activation, control a profile of alternative transcripts independently of its effect on AR target gene transcription. Even fewer studies have addressed the impact of ADT in the form of bicalutamide or enzalutamide on alternative splicing in preclinical CaP models [121, 122]. To move forward with therapeutic strategies aimed at alternative splicing events in CaP, more such studies will need to be done and the relevance of differentially expressed transcripts on CaP cell behavior and response to ADT will need to be resolved. When we consider the impact of taxane-based chemotherapeutics such as docetaxel, which is administered after failure of ADT and increasingly in combination with ADT [160, 161], even less is known. We could not locate any studies that directly assessed the effect of docetaxel on the CaP alternative transcriptome. A few studies did report on alternative splicing events that contribute to CaP’s docetaxel resistance. We already mentioned the FGFR3-S variant that is preferentially expressed in African American (AA) CaP [26] and decreased caspase activity causing resistance to docetaxel-induced apoptosis. Others found that β-Arrestin2 promotes docetaxel resistance of CRPC via hnRNP A1-mediated PKM2 alternative splicing [162]. Upon docetaxel treatment, PC3 cells that overexpress the osteopontin (OPN) b or c isoforms showed higher cell densities, decreases in EMT epithelial cell markers, and upregulated mesenchymal markers compared to cells overexpressing OPNa and controls, suggesting that OPNc or OPNb overexpression in PC3 cells can mediate resistance to docetaxel-induced cell death [163]. To what extent ADT-induced splice variants may impact the response to chemotherapeutics is similarly understudied. Even though docetaxel therapy induces AR-V7 expression in CRPC patients and in CRPC cells after long-term exposure [164], circulating tumor cell (CTC) levels of AR-V7 are associated with resistance to enzalutamide and abiraterone [110], but not with primary resistance to taxane chemotherapy in CRPC [165]. In AR-V7-positive men, taxanes appear to be more efficacious than enzalutamide or abiraterone therapy, whereas in AR-V7-negative men, taxanes and enzalutamide or abiraterone may have comparable efficacy [165].

Another issue relates to how alternative splicing is impacted by interCaP and intraCaP heterogeneity or to what extent it causes it. Indeed, alternative splicing itself could introduce heterogeneity within the CaP cell population causing them to divert away from normal developmental pathways and resulting in altered drug sensitivities. One example that supports this possibility is, as discussed previously in the text, the alternative splicing of *REST* which promotes the emergence of a neuroendocrine phenotype in CRPC cells and drives lineage plasticity [23]. Another example is the alternative splicing of the *ERG* gene in CaP that gives rise to multiple TMPRSS2-ERG fusion transcripts [166]. These transcript variants fall into two categories: type I splice variants encode complete ERG proteins, while type II variants encode a shorter ERG version, lacking the E26 transformation-specific (ETS) domain. Notably, type II splice variants were discovered to be more prevalent in clinical CaP samples. The ratio of type I to type II splice variants has also been linked to clinical features in CaP patients, with a higher ratio correlating with a less favorable outcome [166]. While it has been well-studied that the heterogeneity of TMPRSS2-ERG rearrangements contributing to tumor heterogeneity in CaP [167], the splice variants may add further complexity to it. If this holds true, then targeting ERG splicing may provide promising therapeutic strategy to manage CaP of different origins. The SSO approach that targets ERG’s exon 4 and results in decreased cell proliferation, cell migration and significantly increased apoptosis in in vitro and in vivo model systems [158], supports this possibility. Therefore, contribution of alternative splice variants in the development of CaP tumor heterogeneity needs to be explored further. The increasing application of scRNA-Seq to CaP cell population could theoretically help resolve these questions [168]. Unfortunately, the scRNA-Seq short reads to date are not yet able to detect differences in splicing patterns between cell populations.

Furthermore, inhibiting the activity of SRGs, even those that are overexpressed or mutated in cancer, impacts also non-malignant tissues, which may limit the therapeutic window and applicability. Clinical trials testing compounds such as E7107 in other human diseases have been terminated due to off target effects [169]. Even though it is tempting to assume that their effects on early steps of spliceosome action cause these drugs to completely shut down alternative splicing, there is at least some selectivity to their effects. This selectivity has been linked to RNA sequences having different sensitivities to these compounds, with sequences with stronger base-pairing to U2 snRNA being more resistant [170]. When considering trans-acting approaches, it is important to recognize also that the majority of SRGs or those SRGs most relevant to CaP treatment resistance may either not be druggable or to date have no specific inhibitor. Compounds such as herboxidiene still remain to be tested in CaP despite some efficacy in other human cancers [171].

RNA-based cis-acting approaches have the potential to be more specific and to cause less toxicities but suffer from other limitations. Such strategies require accurate motifs information on key regulatory or binding RNA sites while these sequences are poorly described. To date, it is not yet clear which of the deregulated alternative CaP mRNA transcripts mediate the most relevant biological consequences for disease progression. Because of technical constraints with the short read RNA-sequencing methods that are predominantly used, the most important isoforms may not even be known yet. Unlike small molecule inhibitor-mediated therapeutics, efficient delivery and cellular uptake of SSOs may limit their effective administration.

## Conclusions

The study of alternative splicing in CaP, its molecular determinants, biological consequences and therapeutic potential is an emerging field. Although knowledge has increased over the past few years, there are still some controversies and many unknowns that need to be resolved. It has become clear that alternative splicing events that are controlled by both the major and minor spliceosomes occur with increasing frequency and some stage-specificity during CaP progression. Some, such as AR variants and REST splicing, have already been directly implicated in failure of ADT and emergence of NEPC, which illustrates the relevance of at least some of the identified alternative transcripts for aggressive CaP behavior and disease progression. The biological relevance or implications of most other CaP-specific alternative transcripts is as of yet unknown but will be important to determine. This already raises some more questions. For instance, how best to select transcripts for follow-up studies and how to determine optimal experimental conditions to do so? One approach could be to focus on those transcripts whose differential expression has been linked to poor outcome, but this information is scarce for isoforms whose expression is altered in treatment-resistant CaP. With regard to testing their biological impact, should this be done in cells in which all transcript variants of the same gene are depleted, in isolation or in combination with different ratios of other transcripts of the same gene or other genes… Another caveat relates to how well RNA isoform levels actually correlate with those of protein isoforms. Answering that question may require that more sophisticated mass spectrometry approaches are applied. Increasingly, it is recognized that deregulation of the CaP alternative splicing landscape is accompanied by changes in trans-acting factors [19]. This involves their deregulated expression [19] and, at a lower frequency, somatic mutations [120]. However, which transcripts are controlled by the SRGs that are altered in CaP is yet to be determined, and the extent to which the observed changes in their expression or structural integrity impact SRG-dependent transcriptomes is also unknown. SRG inhibition has shown preclinical therapeutic promise in CaP even though to date, to our knowledge, other than testing HSP90 inhibitors no clinical trials have been performed in CaP (clinicaltrials.gov). Supporting evidence for changes in cis-acting elements that affect splicing at these or surrounding sites (such as SNPs, duplication etc.) is currently weak and the functional consequences of such alterations remain to be validated experimentally. Obtaining such information and additional sequence data will be important to expand the scope of CaP-specific RNA-targeting therapeutic options. Whether CaP-specific RNA isoforms may represent druggable targets themselves is not yet fully explored although there are precedents in other human cancers. For instance, FN1 splicing, which we found to be controlled by CIT in CaP [131] has been associated with colon cancer and splicing of its extracellular domain proposed as therapeutic strategy [172, 173]. Although splice variants can give rise to neoantigens that have been linked with response to immunotherapies in other malignancies, even late-stage CaPs are notoriously immunocold [174], questioning the usefulness of such approaches to exploit alternative splicing for therapy. At least conceptually, other possibilities for therapeutic intervention may exist. For instance, our recent work showed that CIT controls a pattern of ~900 transcripts that is enriched in NEPC and CRPC [131], suggesting that CaP’s alternative splicing landscape may be broken down into fractions that are coordinately regulated and could eventually be targeted for therapy simultaneously.
